# Supplementary material for: Atomistic modeling of liquid-liquid phase equilibrium explains dependence of critical temperature on γ-crystallin sequence
Source: Commun Biol. 2023 Aug 29;6:886. doi: 10.1038/s42003-023-05270-7 (PMC10465548; doi:10.1038/s42003-023-05270-7)
Supplement: Supplementary file 2 — Supplementary Information [file 42003_2023_5270_MOESM2_ESM.pdf]

Atomistic Modeling of Liquid-Liquid Phase Equilibrium Explains Dependence of Critical Temperature on γ-Crystallin Sequence

Sanbo Qin[a] and Huan-Xiang Zhou[a,b,]*

[a]Department of Chemistry and [b]Department of Physics, University of Illinois Chicago, Chicago, IL 60607, USA

*To whom correspondence should be addressed. E-mail: hzhou43@uic.edu.

Supplementary Information

Supplementary Note 1

Here we place our FMAP-based approach in the broad context of various approaches used in calculating binodals of protein phase separation, and present a critical analysis of our approach.

Representation of the protein solution. Four types of approaches that differ greatly in the level of realism in representing protein solutions, with commensurate differences in computational cost, have been used to calculate binodals of protein liquid-liquid phase separation (Supplementary Fig. 2). In the first three approaches, solvent is only modeled implicitly. At the low end of the realism / computational cost spectrum are studies where proteins are represented as rigid spheres with interaction sites on the surface [1-3]. There are many reports at the next step up, where proteins are represented at the coarse-grained (CG) level and allowed to be flexible [4-12]. This approach is usually used for the study of intrinsically disordered proteins. A different compromise, where proteins are represented at the all-atom (AA) level but kept rigid, is better suited for the study of structured proteins. We have demonstrated the feasibility of this approach when the chemical potential was calculated by the FMAP method [13]. Finally, the most realistic but also most expensive approach is one where both protein and solvent molecules are represented at the AA level and allowed to be flexible. We have reported the binodal of a tetrapeptide using this approach [12]. AA explicit-solvent simulations have also been used to calculate potentials of mean between pairs of amino acids or for the dissociation of a short peptide from a tetrameric assembly to provide mechanistic insight into the effects of salt and β-sheet clusters on phase separation [14-16].

Our goal here is to model the sequence dependence of the binodals for the phase separation of structured proteins. For quantitative predictions of sequence dependence, an AA representation of the proteins is required. However, it is also clear that a full AA

model with molecular flexibility is not feasible. We thus chose the next best level, i.e., an AA representation but with a rigid treatment of the proteins along with implicit solvent.

Interaction energy function. The next issue to be determined is the energy function. Energy functions for implicit-solvent models are generally written as the sum of molecular mechanics (MM) terms and solvation ("solv") terms:

$$U = U_{\mathrm{MM}} + U_{\mathrm{solv}} \qquad \text{[S1]}$$

The MM terms typically include van der Waals interactions and Coulomb interactions, whereas the solvation terms include nonpolar solvation and polar solvation. For example, in the MM/GBSA model [17], the polar solvation term is determined by the generalized Born model, whereas the nonpolar solvation term is the product of the solvent accessible surface area and a constant (equivalent to the protein-solvent interfacial tension).

Even with the protein treated as rigid and solvent treated implicitly, calculating the chemical potential for determining the binodal for an AA representation of the protein is still very expensive. This calculation was made feasible by the FMAPμ method [13]. The key idea behind this method is to express interaction energy terms as correlation functions, which are then evaluated via fast Fourier transform (FFT). The FFT route places certain restrictions on the forms of the interaction energy terms. Partly on account of this restriction, we combined the van der Waals interaction and nonpolar solvation terms and assumed a scaled Lennard-Jones potential for their combined contribution (Eqs [4] and [6]). Likewise, we combined the Coulomb interaction term and polar solvation into a scaled Debye-Hückel potential (Eqs [4] and [7]).

In comparison, the more sophisticated Rosetta energy function [18] comprises weighted terms including a Lennard-Jones potential, a desolvation term, an electrostatic term using a distance-dependent dielectric constant, an orientation-dependent hydrogen bonding term, and statistical potentials for backbone and sidechain torsional preferences.

Factors that affect FMAP-based calculation results. The components in our procedure for calculating the binodal, in particular the FMAPμ method, have been carefully validated to ensure numerical accuracy [19]. Still, other factors can affect the calculation results and their accuracy. One is the parameterization of the interaction energy function and another is the configurational sampling used for chemical potential calculation.

Regarding parameterization, two notable parameters are the scaling constants, s_1 and s_2, for the nonpolar attraction and electrostatic terms (see Eq [4]). In a 2019 paper [20], we carried out this parameterization using experimental results for the second virial coefficients of proteins. Here we have also explored the effects of these two scaling constants (Supplementary Fig. 7). The default values for s_1 and s_2 were set to 0.16 and 1.6, respectively. The resulting second virial coefficient for γB agrees well with experimental data (Fig. 4A inset). However, there are also other combinations of s_1 and s_2, by either giving a higher weight to the nonpolar attraction but with a commensurate reduction in the weight for the electrostatic term or vice versa, that produce a similar agreement (Supplementary Fig. 7a). For all these combinations of s_1 and s_2, a higher T_c is predicted for γF than for γB (Supplementary Fig. 7b).

As for configurational sampling, we have taken great pains to achieve convergence. As stated in the main text, each μ_{ex} calculation is based on 1.57464×10^{14} interaction energies. So within the limitations of the model (i.e., rigid proteins and implicit solvent), the calculated results are accurate and the conclusions drawn are robust.

Possible treatment of protein flexibility in binodal calculations on all-atom models. We are not aware of any binodal calculations based on AA flexible protein models with implicit solvent (hatched box in Supplementary Fig. 2). Full treatment of flexibility seems extremely challenging at this time, but it might be feasible to include a limited extent of flexibility. One possibility is to allow the protein molecules to sample a

preselected library of structures. This possibility was explored in Monte Carlo simulations of many-protein systems [21]. As an initial step toward sampling multiple conformations in chemical potential calculations for dense protein solutions, we tested conformational sampling in calculations of the second virial coefficient. The preselected library consisted of all the high-resolution (2.3 Å or better) X-ray structures in the Protein Data Bank (PDB) for a given γ-crystallin. For example, there are four such structures available for rat γE. As shown in Fig. 7, the $B_2/V_{\rm st}$ values calculated from the individual PDB structures range from -1.8 to -2.4. This range demonstrates that input structures have nonnegligible effects on the calculated B_2 results. A simple way to remove the uncertainty in B_2 arising from the choice of input structure is to assume that the protein samples all these structures with equal probability. The resulting B_2, by averaging values from the $4 \times 4 = 16$ binary combinations of structures, is -2.0 ± 0.2 (Supplementary Fig. 11).

The structures in the preselected library could have both global changes and local changes (e.g., sidechain rotation), but the number of structures is limited by the amount of computational time that can be afforded. In the foregoing B_2 calculations, the computational time is proportional to the number of structure pairs included. For proteins like γ-crystallins that do not show any sign of large conformational changes (e.g., as evidenced by close similarities between multiple X-ray structures), it may be more important to sample sidechain rotations that are induced by intermolecular interactions in a dense protein solution. We thus tested for the effects of sidechain repacking using RosettaDock [22], on the 1,000 lowest interaction energy poses from FMAPB2 calculations. We obtained the Rosetta interaction energies either after a simple energy minimization (Supplementary Fig. 10a) or after subsequent sidechain repacking (Supplementary Fig. 10b). As expected, the γF poses have lower FMAP interaction energies than the γB poses, as illustrated by an energy gap of 0.20 kcal/mol at CDF = 100 (Supplementary Fig. 10c). Interestingly, the gap widens to 0.59 kcal/mol according to the Rosetta energy function,

and widens even further to 0.92 kcal/mol upon sidechain repacking (Supplementary Fig. 10d). These results demonstrate two points. First, a more sophisticated energy function like Rosetta may more accurately capture the gap in T_c between γF and γB. Second, sidechain repacking could contribute to the gap in T_c.

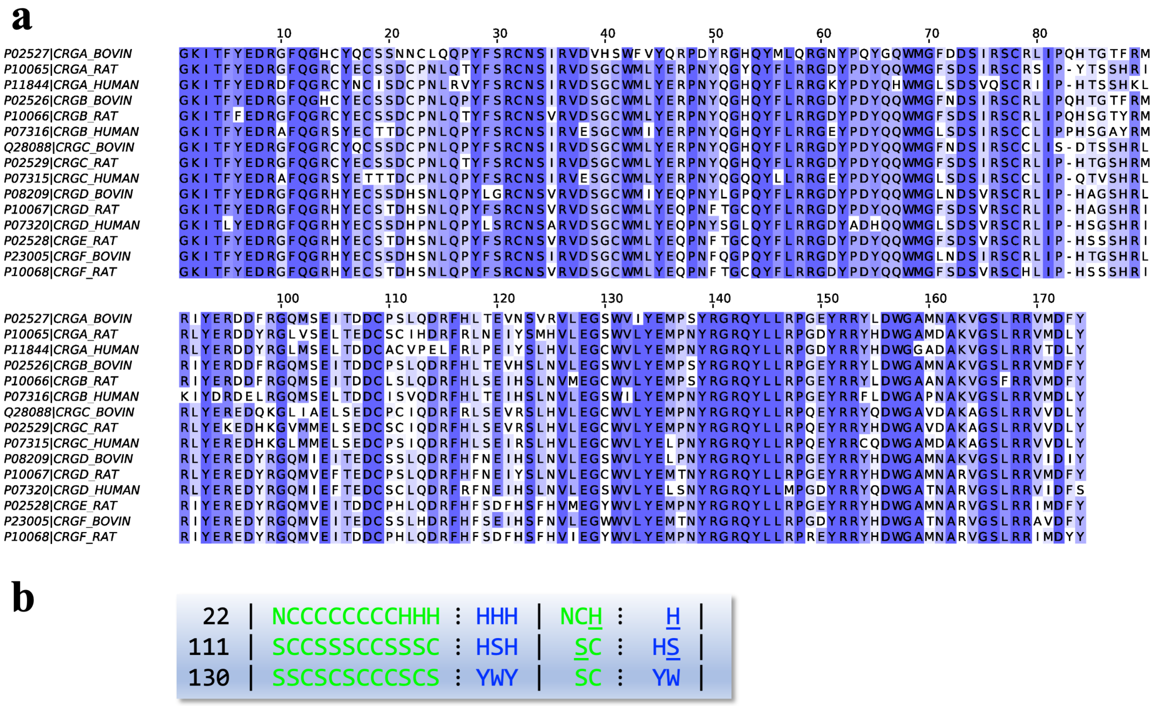

Supplementary Figure 1. Sequence alignment of γ-crystallins. (a) The names and sequences of all entries of bovine, human, and rat γ-crystallins in UniProt (https://www.uniprot.org/), aligned by ClustalW [23] and visualized using Jalview (https://www.jalview.org/). Residue numbers are according to bovine γB. (b) Sequence comparison at three positions showing the largest Grantham distances between the low and high-T_c γ-crystallins (Fig. 5). Columns 2 and 3 display the amino acids at the same position for the 12 low-T_c and 3 high-T_c γ-crystallins. Columns 4 and 5 display a nonredundant list of amino acids within the low-T_c and high-T_c groups; amino acids that occur in both groups are underlined.

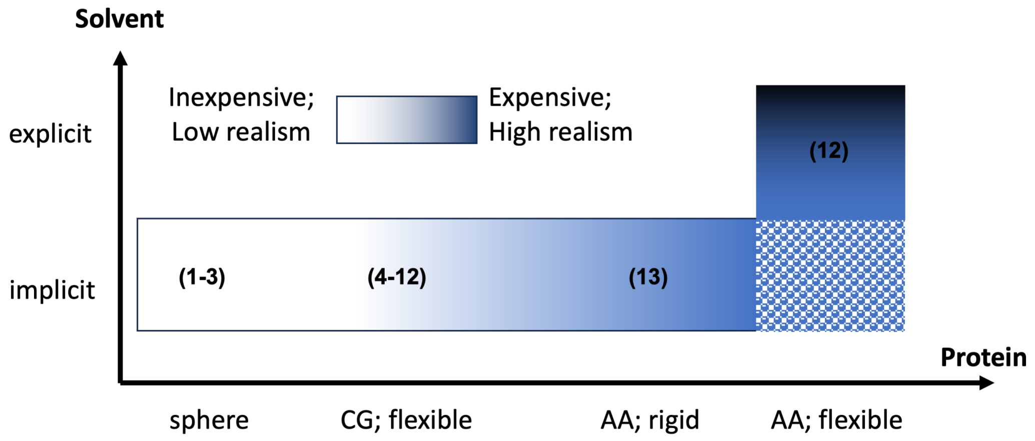

Supplementary Figure 2. Illustration of the different approaches used to calculate binodals of protein liquid-liquid phase separation. Studies that reported binodals are cited by reference numbers. The intensity of the blue shading increases with the realism and computational cost of the approach. We are not aware of any study reporting binodals for proteins treated at the AA level and allowed to be flexible (hatched region).

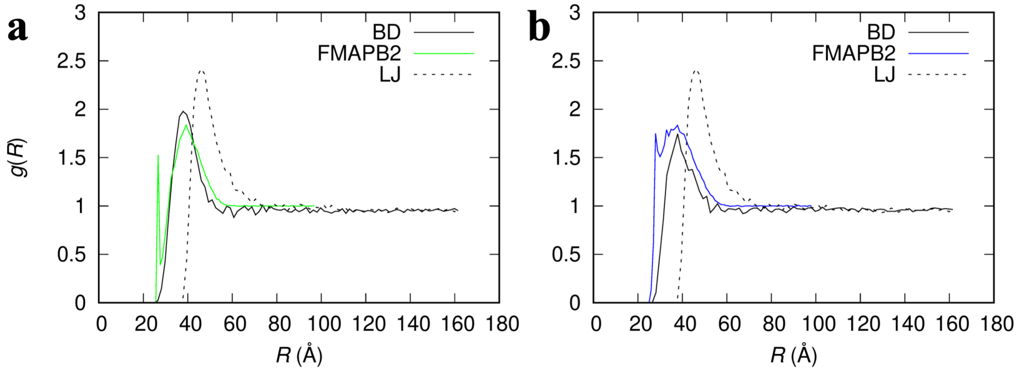

Supplementary Figure 3. Comparison of pair distribution functions. (a) γB. (b) γF. The "BD" trace is by counting pairs in distance bins in BD simulations at 300 K and the lowest concentration (31 mg/ml). The FMAPB2 trace is from averaging the Boltzmann factor of the pair interaction energy at 298 K in distance bins and equating the average Boltzmann factor to $g(R)$. The "LJ" trace is from counting pairs in distance bins in simulations of Lennard-Jones particles at $k_B T/\varepsilon = 1$ and $N = 30$ reported in our 2016 study [13]. The Lennard-Jones σ parameter was scaled to 40.5 Å, which is close to the diameter of a sphere with the steric volume $V_{st} \equiv B_2^{st}/4$ of γB.

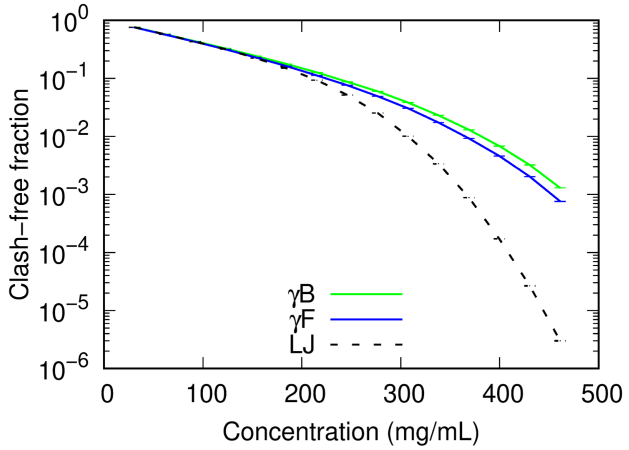

Supplementary Figure 4. The clash-free fraction when inserting a test protein into a protein solution. The γB and γF results were calculated by FMAPμ and averaged over 2,000 BD configurations and 500 test-protein orientations. The trace labeled "LJ" was calculated similarly on configurations generated by replacing Lennard-Jones particles with an all-atom structure of γB at an arbitrary orientation, as reported in our 2016 study [13] (10 test-protein orientations). The simulations of Lennard-Jones particles were done at a reduced temperature $k_B T/\varepsilon = 1$ and at the same total volume and particle numbers (N = 30 to 450) as the BD simulations of γB and γF. Error bars were estimated by the blocking method.

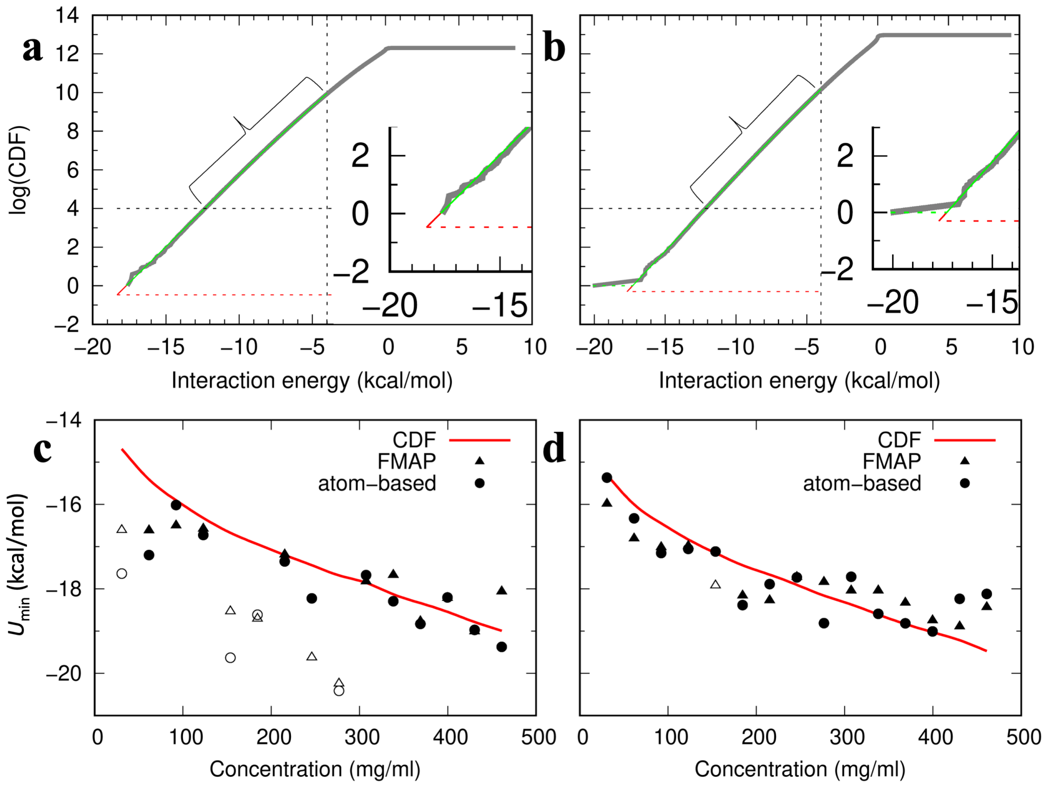

Supplementary Figure 5. Correction of the chemical potential by modeling the cumulative distribution function (CDF) in the low interaction energy region. (a) CDF collected from the allowed insertions in FMAPμ calculations over 2,000 BD configurations and 500 test-protein orientations, shown as gray curve for γB at 369 mg/ml. The bracketed portion, bounded from below at CDF = 10^4 (black horizontal line) and from above at U = -4 kcal/mol (black vertical line), was locally fit to a linear function. The fit function is extrapolated to the lower bound given by Eq [18] (red horizontal line). The extrapolation is shown in green down to CDF = 1 and in red beyond. A zoomed version of the lower left corner is shown in the inset. (b) Corresponding results for γB at 277 mg/ml. In contrast to (a) where the raw CDF stays close to the extrapolation all the way down to CDF = 1, a single configuration with an outlying low interaction energy (= -20.1 kcal/mol) markedly shifts the raw CDF away from the extrapolation. (c) Minimum interaction energies of γB at various concentrations. Symbols display results

obtained according to Eq [17], illustrated in Supplementary Fig. 6; triangles are from FMAPμ calculations whereas circles from atom-based calculations. Open symbols are for cases with an outlying lowest interaction energy, identified by a gap of at least 1.0 kcal/mol between that energy and the one from extrapolating the CDF fit function to CDF = 1, as illustrated in the panel (b) inset. Filled symbols are for cases without such outliers. The curve displays the results predicted by Eq [18]. (d) The corresponding results for γF.

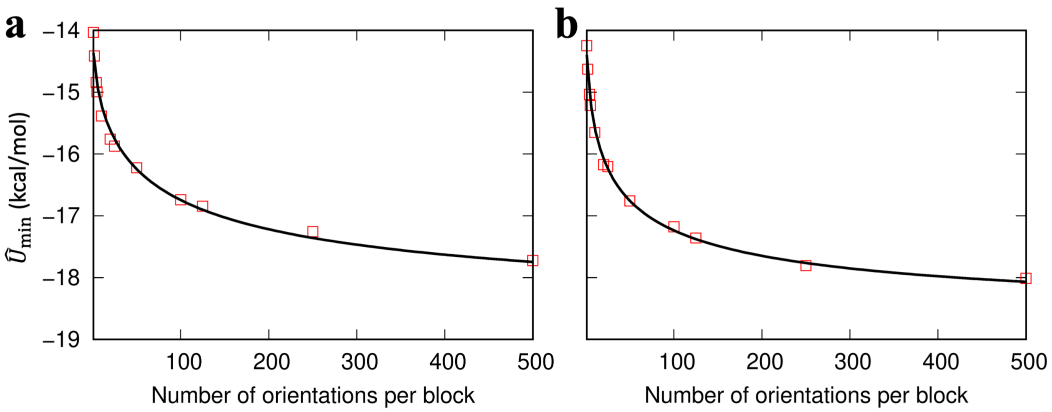

Supplementary Figure 6. The dependence of the mean lowest interaction energy on the block size, i.e., the number of test-protein orientations per block. Results are for (a) γB and (b) γF at 369 mg/ml. The symbols display results from the atom-based method; the curves display the fit to Eq [17].

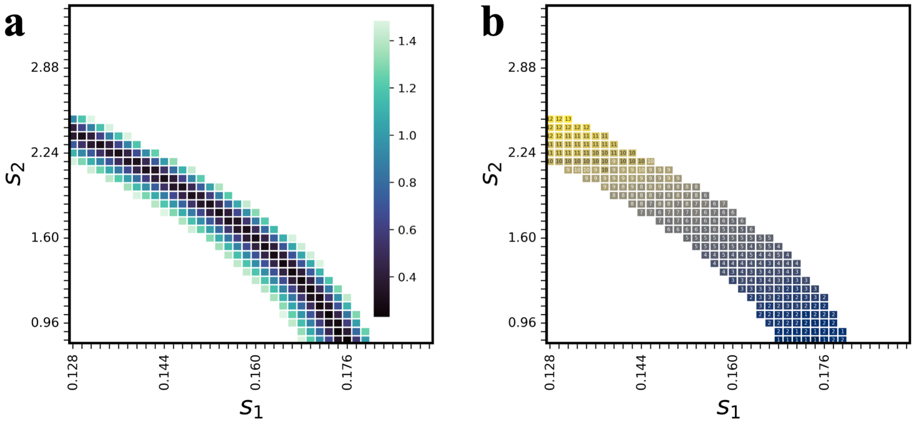

Supplementary Figure 7. Effects of varying the scaling factors s_1 and s_2 in the energy function. (a) Root-mean-square-error (RMSE) of calculated B_2 values relative to the corresponding experimental B_2 data for γB [24]. A direct comparison of calculated and experimental values at the default s_1 (= 0.16) and s_2 (= 1.6) is shown in Fig. 4 inset. The scale bar is for RMSE in units of the steric volume (V_{st}) of γB. The displayed pixels represent "acceptable" s_1-s_2 combinations, i.e., those resulting in RMSE/V_{st} < 1.5; the latter value is the error magnitude of the experimental data (Fig. 4 inset). (b) The difference in T_c between γF and γB in the acceptable region of the parameter space.

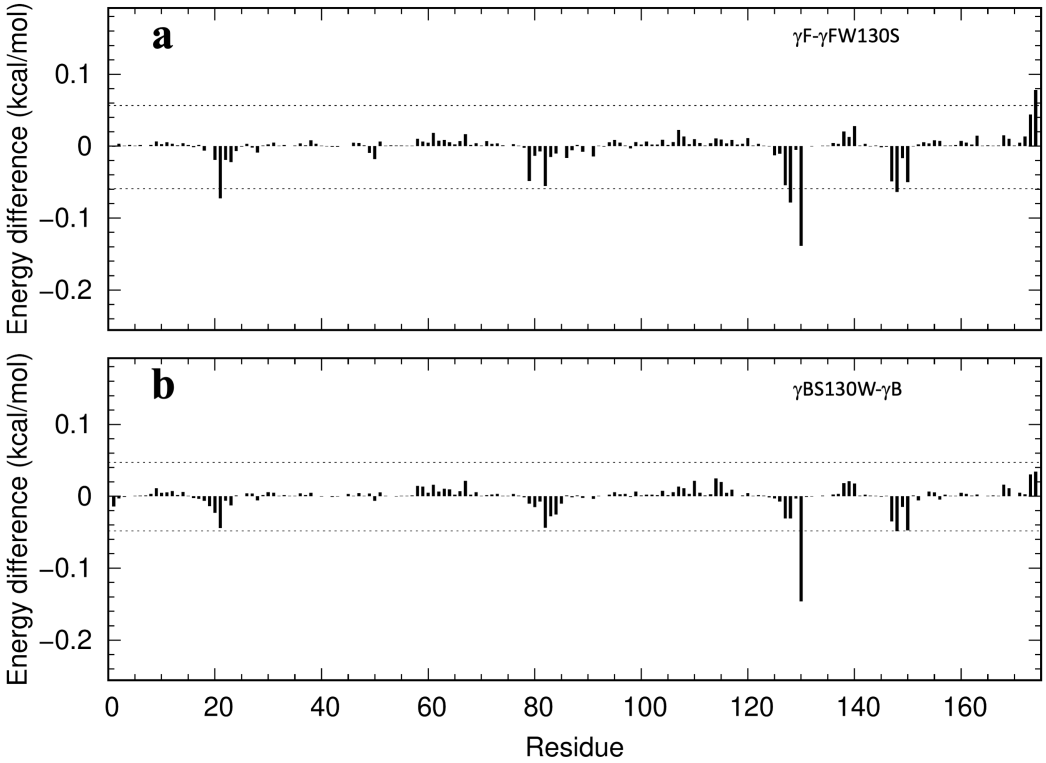

Supplementary Figure 8. Difference in residue-specific decomposed pair interaction energies. (a) The difference with the γFW130S results subtracted from the γF results. (b) The difference with the γB results subtracted from the γBS130W results.

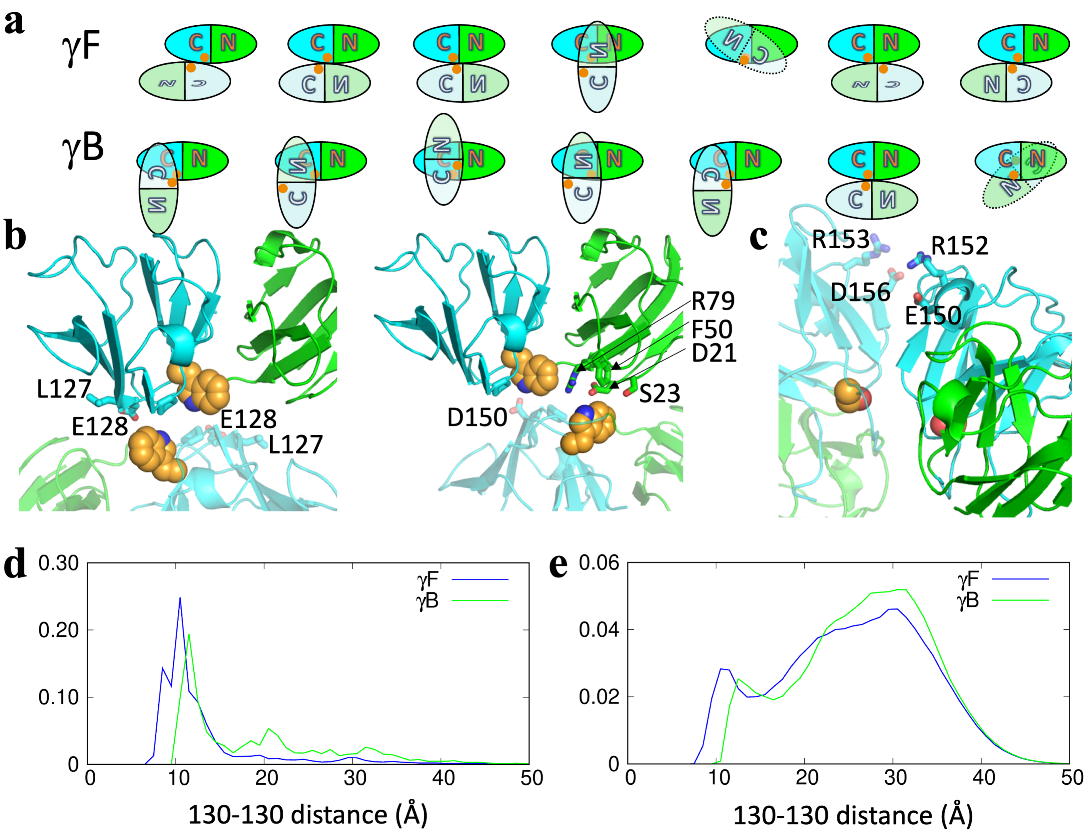

Supplementary Figure 9. Different tendencies of γF Trp130 and γB Ser130 to be buried in binary interfaces. (a) Illustration of the arrangements between monomers in the seven large-cluster representatives of γF or γB. "C" and "N" label the N and C-terminal domains; reflected, slanted, and upside-down letters indicate self-rotation of a monomer. Orange circles represent Trp130 or Ser130 residues. (b) Cross-interface contacts in the representative poses of large cluster 1 and large cluster 3 of γF. (c) Cross-interface contacts in the representative pose of large cluster 1 of γB. (d) Distribution functions of Trp130-Trp130 distances in γF pairs (blue curve) and Ser130-Ser130 distances in γB pairs (green curve). Inter-residue distances were calculated for all poses with interaction energies < -6 kcal/mol, each weighted by the Mayer function (T = 25 °C). (e) Distributions of 130-130 distances in BD simulations of γF and γB. In each snapshot, one 130-130 distance was obtained for each protein molecule, i.e., the shortest one from a neighboring molecule. The distribution function of 130-130 distances was then averaged

over 2,000 snapshots from γF or γB simulations at $N = 390$ (corresponding to 400 mg/ml).

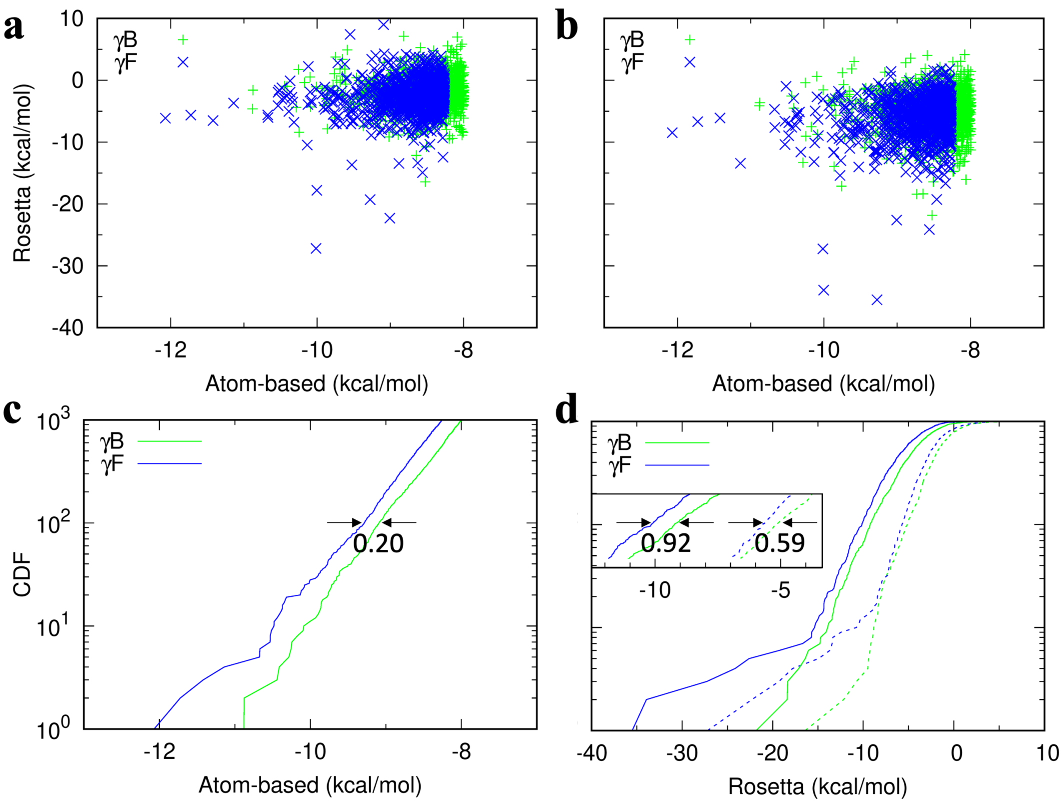

Supplementary Figure 10. Effects of applying the Rosetta energy function and sidechain repacking. Results were obtained by using RosettaDock on the 1,000 lowest interaction energy poses of γB or γF from FMAPB2 calculations. (a) Rosetta interaction energies after a simple energy minimization, paired with those calculated by the atom-based method using the FMAP energy function. (b) Corresponding results from RosettaDock after applying sidechain repacking. (c) CDFs of the FMAP interaction energies for γB and γF. (d) CDFs of the Rosetta interaction energies after energy minimization or upon subsequent sidechain repacking. Double arrowheads indicate the energy gaps between γB and γF at CDF = 100; numbers are the energy gaps in kcal/mol.

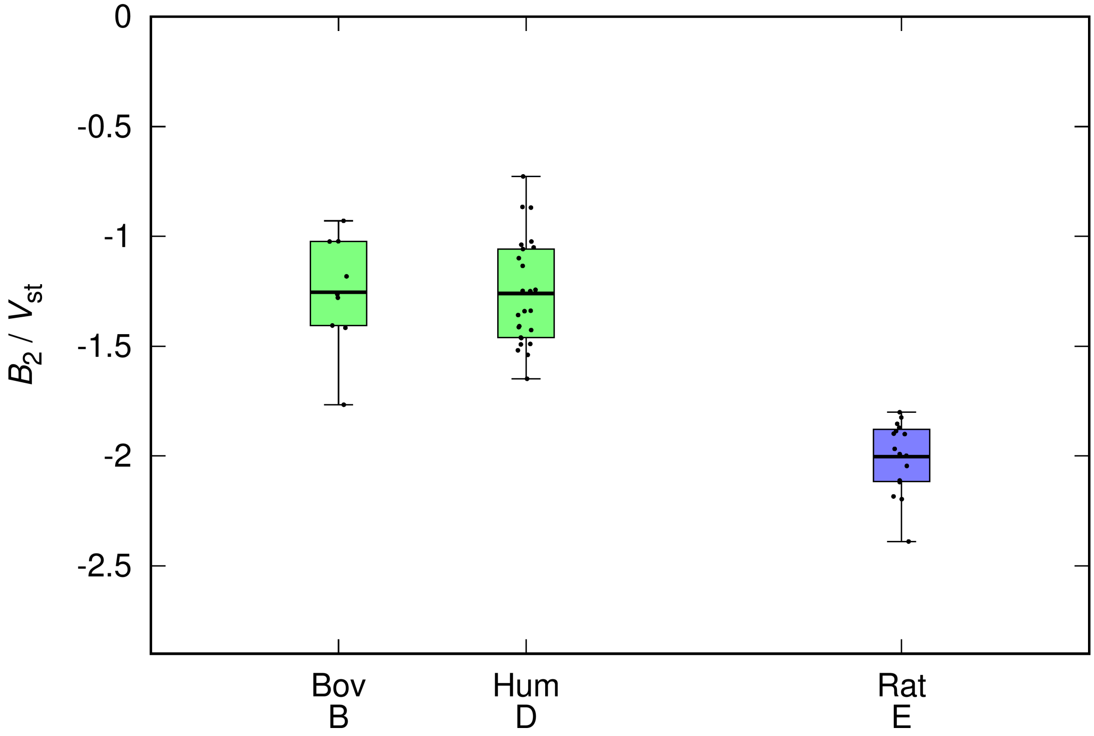

Supplementary Figure 11. Second virial coefficients calculated by averaging over a preselected library of *n* structures. Points, shifted in the horizontal direction by arbitrary amounts to minimize overlap, display the individual results from *n* × *n* binary combinations of structures; black bar displays the mean value, while other parts of the box plot have the usual meanings (i.e., minimum and maximum as well as first and last quartiles). *n* = 3, 5, and 4 for bovine γB, human γD, and rat γE, respectively. Structures are from the PDB, with entry names shown in Fig. 7.

Supplementary References

1. Kastelic, M., Kalyuzhnyi, Y. V., Hribar-Lee, B., Dill, K. A., Vlachy, V. Protein aggregation in salt solutions. *Proc Natl Acad Sci U S A* **112**, 6766-6770 (2015).
2. Nguemaha, V., Zhou, H. X. Liquid-Liquid Phase Separation of Patchy Particles Illuminates Diverse Effects of Regulatory Components on Protein Droplet Formation. *Sci Rep* **8**, 6728 (2018).
3. Ghosh, A., Mazarakos, K., Zhou, H. X. Three archetypical classes of macromolecular regulators of protein liquid-liquid phase separation. *Proc Natl Acad Sci U S A* **116**, 19474-19483 (2019).
4. Das, S., Lin, Y.-H., Vernon, R. M., Forman-Kay, J. D., Chan, H. S. Comparative roles of charge, π, and hydrophobic interactions in sequence-dependent phase separation of intrinsically disordered proteins. *Proc Natl Acad Sci U S A* **117**, 28795-28805 (2020).
5. Dignon, G. L., Zheng, W., Kim, Y. C., Best, R. B., Mittal, J. Sequence determinants of protein phase behavior from a coarse-grained model. *PLoS Comput Biol* **14**, e1005941 (2018).
6. Dignon, G. L., Zheng, W., Best, R. B., Kim, Y. C., Mittal, J. Relation between single-molecule properties and phase behavior of intrinsically disordered proteins. *Proc Natl Acad Sci U S A* **115**, 9929-9934 (2018).
7. Espinosa, J. R., Joseph, J. A., Sanchez-Burgos, I., Garaizar, A., Frenkel, D., Collepardo-Guevara, R. Liquid network connectivity regulates the stability and composition of biomolecular condensates with many components. *Proc Natl Acad Sci U S A* **117**, 13238-13247 (2020).
8. Joseph, J. A., *et al.* Physics-driven coarse-grained model for biomolecular phase separation with near-quantitative accuracy. *Nat Comput Sci* **1**, 732-743 (2021).

9. Mazarakos, K., Zhou, H. X. Macromolecular regulators have matching effects on the phase equilibrium and interfacial tension of biomolecular condensates. *Protein Sci* **30**, 1360-1370 (2021).

10. Tejedor, A. R., Garaizar, A., Ramírez, J., Espinosa, J. R. 'RNA modulation of transport properties and stability in phase-separated condensates. *Biophys J* **120**, 5169-5186 (2021).

11. Tesei, G., Schulze, T. K., Crehuet, R., Lindorff-Larsen, K. Accurate model of liquid–liquid phase behavior of intrinsically disordered proteins from optimization of single-chain properties. *Proc Natl Acad Sci U S A* **118**, e2111696118 (2021).

12. Mazarakos, K., Prasad, R., Zhou, H. X. SpiDec: Computing binodals and interfacial tension of biomolecular condensates from simulations of spinodal decomposition. *Front Mol Biosci* **9**, 1021939 (2022).

13. Qin, S., Zhou, H. X. Fast Method for Computing Chemical Potentials and Liquid-Liquid Phase Equilibria of Macromolecular Solutions. *J Phys Chem B* **120**, 8164-8174 (2016).

14. Krainer, G.*, et al.* Reentrant liquid condensate phase of proteins is stabilized by hydrophobic and non-ionic interactions. *Nat Commun* **12**, 1085 (2021).

15. Garaizar, A.*, et al.* Aging can transform single-component protein condensates into multiphase architectures. *Proc Natl Acad Sci U S A* **119**, e2119800119 (2022).

16. Tejedor, A. R.*, et al.* Protein structural transitions critically transform the network connectivity and viscoelasticity of RNA-binding protein condensates but RNA can prevent it. *Nat Commun* **13**, 5717 (2022).

17. Gohlke, H., Kiel, C., Case, D. A. Insights into protein-protein binding by binding free energy calculation and free energy decomposition for the Ras-Raf and Ras-RalGDS complexes. *J Mol Biol* **330**, 891-913 (2003).

18. Alford, R. F., *et al.* The Rosetta All-Atom Energy Function for Macromolecular Modeling and Design. *J Chem Theory Comput* **13**, 3031-3048 (2017).

19. Qin, S., Zhou, H. X. Further Development of the FFT-based Method for Atomistic Modeling of Protein Folding and Binding under Crowding: Optimization of Accuracy and Speed. *J Chem Theory Comput* **10**, 2824-2835 (2014).

20. Qin, S., Zhou, H. X. Calculation of Second Virial Coefficients of Atomistic Proteins Using Fast Fourier Transform. *J Phys Chem B* **123**, 8203-8215 (2019).

21. Majumdar, B. B., Prytkova, V., Wong, E. K., Freites, J. A., Tobias, D. J., Heyden, M. Role of Conformational Flexibility in Monte Carlo Simulations of Many-Protein Systems. *J Chem Theory Comput* **15**, 1399-1408 (2019).

22. Gray, J. J., *et al.* Protein–Protein Docking with Simultaneous Optimization of Rigid-body Displacement and Side-chain Conformations. *J Mol Biol* **331**, 281-299 (2003).

23. Thompson, J. D., Gibson, T. J., Higgins, D. G. Multiple sequence alignment using ClustalW and ClustalX. *Curr Protoc Bioinformatics* **Chapter 2**, Unit 2 3 (2002).

24. Bucciarelli, S., Mahmoudi, N., Casal-Dujat, L., Jehannin, M., Jud, C., Stradner, A. Extended Law of Corresponding States Applied to Solvent Isotope Effect on a Globular Protein. *J Phys Chem Lett* **7**, 1610-1615 (2016).

Supplementary Movie 1. A 360° view of Fig. 6a.

Supplementary Movie 2. A 360° view of Fig. 6b.
