# Supplementary material for: Atomistic modeling of liquid-liquid phase equilibrium explains dependence of critical temperature on γ-crystallin sequence
Source: Commun Biol. 2023 Aug 29;6:886. doi: 10.1038/s42003-023-05270-7 (PMC10465548; doi:10.1038/s42003-023-05270-7)
Supplement: Supplementary file 3 — Description of Supplementary Materials [file 42003_2023_5270_MOESM3_ESM.docx]

**Description of Additional Supplementary Files**

File name: Supplementary Movie 1

Description: A 360° view of Fig. 6a.

File name: Supplementary Movie 2

Description: A 360° view of Fig. 6b.
